# The role of the Wnt/β-catenin signaling pathway in the proliferation of gold nanoparticle-treated human periodontal ligament stem cells

**DOI:** 10.1186/s13287-018-0954-6

**Published:** 2018-08-09

**Authors:** Chen Li, Zhuoquan Li, Yan Zhang, Abdel Hamid Fathy, Min Zhou

**Affiliations:** 1 Department of Endodontics, School & Hospital of Stomatology, Tongji University, Shanghai Engineering Research Center of Tooth Restoration and Regeneration, Shanghai, 200072 China; 2 The Institute for Translational Nanomedicine, Shanghai East Hospital, The Institute for Biomedical Engineering & Nano Science, Tongji University School of Medicine, Shanghai, 200092 China; 3 Department of Basic Science, School & Hospital of Stomatology, Tongji University, Shanghai Engineering Research Center of Tooth Restoration and Regeneration, Shanghai, 200072 China; 4 Tongji University School of Medicine, Shanghai, 200092 China; 5 Department of Periodontics, School & Hospital of Stomatology, Tongji University, Shanghai Engineering Research Center of Tooth Restoration and Regeneration, Shanghai, 200072 China

**Keywords:** Wnt/β-catenin signaling pathway, Human periodontal ligament stem cell proliferation, Gold nanoparticle, shRNA, siRNA

## Abstract

**Background:**

Several studies have confirmed that gold nanoparticles (AuNPs) of specific concentration and size exert a boosting effect on cell proliferation; however, the mechanism through which this effect occurs remains unknown. This study explores the canonical Wnt signaling pathway in AuNP promotion of human periodontal ligament stem cell (hPDLSC) proliferation.

**Methods:**

MTS was employed to evaluate hPDLSC proliferation. The interference of *LRP5* and β-catenin was steered by shRNA plasmids and siRNA, respectively, at which point the expression of *MYC*, *CCND1*, *AXIN2*, and *POU5F1* had been estimated via real-time PCR. The expressions of LRP5 and β-catenin were detected via western blot assay.

**Results:**

The proliferation of hPDLSCs treated with 60 nm AuNPs at 56 μM was clearly elevated. In contrast, β-catenin siRNA significantly decreased cell viability. The LRP5 shRNA plasmid did not consistently impact cells. The expressions of these four genes downstream of the Wnt/β-catenin signaling pathway were not significantly overexpressed in response to the interference of shRNA plasmid/siRNA with the treatment of AuNPs.

**Conclusions:**

These results suggest that the Wnt/β-catenin signaling pathway plays a significant role in the process of AuNP promotion of hPDLSC proliferation.

## Background

As a result of the rapid development of tissue engineering, proliferation of human periodontal ligament stem cells (hPDLSCs) [1] as seed cells both in vitro and in vivo is a newly emerging technique. Gold nanoparticles (AuNPs) have been used for many scientific applications during the last two decades. Their specific chemical and physical-dependent properties highlight them as very promising agents in many fields such as drug delivery, targeting, imaging [2], and even vaccination [3]. Recently, multiple ex-vivo studies have shown that a specific size and concentration of AuNPs have a promoting effect on cell proliferation [4, 5], and it has been confirmed that the cytotoxicity of AuNPs is a size-dependent, concentration-dependent, and time-dependent effect [6–9]. Consequently, gold nanoparticles have an extensive application prospect in tissue engineering.

Our previous experiments confirmed that 60 nm AuNPs at a concentration of 56 μM significantly promoted the proliferation of hPDLSCs, while exerting no apparent side effects on the differentiation potential of cells. However, another study reported that AuNPs mainly promote the differentiation of human adipose-derived mesenchymal stem cells (MSCs) in osteogenesis via various signaling pathways [10]. Moreover, AuNPs of different sizes can affect the physical processes of cells via diverse signaling pathways. However, the mechanism by which AuNPs promote the proliferation or differentiation of MSCs remains unclear. Therefore, the molecular mechanism by which AuNPs promote MSC proliferation remains to be explored.

Wnt/β-catenin signaling is crucial for regulating embryonic development and cell proliferation as well as for the determination of the cell fate [11]. Proliferation and growth of stem cells are premises of the mass differentiation of cells needed to form tissues and ultimately organisms. This process is established on canonical Wnt signaling, which increases nuclear and cytoplasmic β-catenin and initiates transcriptional activators such as *MYC* and *CCND1* [12]. Recently, Wnt signaling has been implicated in the control of MSC differentiation, including osteogenic, chondrogenic, adipogenic, and myogenic differentiation [10, 13].

Here, an in-vitro study was conducted to investigate the role of the Wnt/β-catenin signaling pathway for the proliferation of hPDLSCs treated with AuNPs (60 nm at 56 μM). LRP5 and β-catenin were interfered with by shRNA and siRNA, respectively. Changes in the expression of Wnt-related genes (*MYC*, *CCND1*, *AXIN2*, and *POU5F1*) were evaluated via real-time PCR. The results indicate that AuNPs promote the proliferation of hPDLSCs, which is likely a result of the Wnt/β-catenin signaling pathway.

## Methods

### hPDLSC culture

Premolars from teenagers 12 and 13 years old, which had been extracted for orthodontic reasons, were freshly obtained from the Oral Surgery Department, Hospital of Stomatology, Tongji University with informed consent. The teeth were rinsed three times with PBS complemented with Antibiotic–Antimycotic solution. The periodontal ligament tissue in the middle third of the root was carefully removed with a surgical scalpel. The tissue was minced and enzymatically digested using type I collagenase (1%; Gibco) and Dispase II (2.5%; Roche) for 1 h at 37 °C. A suspended cell solution was passed through a 70-mm cell strainer to prepare a single cell suspension. The cells were centrifuged and rinsed with PBS and the obtained cell pellet was resuspended and cultured in alpha-modified minimum essential medium (α-MEM; Gibco) with 10% FBS (Gibco) at 37 °C in 5% CO_2_ in humidified air, until the cells proliferated and reached confluence. Human periodontal ligament cells (hPDLCs) were supplemented with an immunofluorescence identification using vimentin and cytokeratin. STRO-1^+^ hPDLCs were sorted via flow cytometry (FCM) and preliminary identification of the stem cell characteristics was achieved via a plate clone formation assay, multidirectional induction as osteoblasts, and adipocytic differentiation.

### Characterization of 60 nm AuNPs

The 60 nm AuNPs were acquired from British Biocell International Company (BBI, UK). The final concentration of AuNPs was adjusted with α-MEM medium. The AuNPs were characterized using both a UV–Vis spectrophotometer (Cary 50; Varian) and a Zetasizer (Nano-ZS90; Malvern).

### shRNA plasmid and siRNA interference

For both shRNA plasmid and siRNA interference, cells were seeded at a density of 2 × 10^6^ cells per well in six-well culture plates and were cultured until 50–70% confluence. Cells were transfected according to an established shRNA transfection protocol [14]. After incubation for 6 h at 37 °C in 5% CO_2_ in humidified air, cells were cultured overnight with 1 ml α-MEM (Gibco) containing 20% FBS and 1% Antibiotic–Antimycotic. The medium was carefully removed and cells were washed with PBS. After culture for 3 h with AuNPs (60 nm at 56 μM), hPDLCs were washed with PBS. The culture medium was replaced by α-MEM, and the cells were cultured for 48 h.

### Cell viability assay

To measure cell viability, hPDLCs were interfered with by either shRNA plasmid or siRNA, and were then plated in triplicate in 96-well tissue culture-treated plates at a density of 1000 cells per well and incubated overnight. The medium was carefully removed and cells were washed with PBS. The 60 nm AuNPs were diluted with fresh serum-free α-MEM medium at a concentration of 56 μM and were added to the hPDLCs. After 2 h, the medium was aspirated and replaced with full medium. Cells without AuNPs were used as control; wells without AuNPs and cells were used as blanks for spectrophotometric readings. MTS (20 μl; Promega) was added to each well at 0, 24, 48, 72, and 96 h. After incubation at 37 °C for 2 h, the optical density (OD) value for each well was measured spectrophotometrically at 490 nm.

The viability assay (Promega) was conducted according to the manufacturer’s instructions. Results were reported as percentage relative fold changes for each group and were internally normalized to the OD reading at 0 h.

### Real-time PCR analysis

After inducing interference with siRNA or shRNA (with or without AuNPs), hPDLSCs were washed with PBS. Total RNA was isolated using Trizol reagent following the manufacturer’s instructions and the RNA concentration was measured spectrophotometrically (Eppendorf Biophotometer, Germany). First-strand cDNA was then synthesized using a PrimeScript First Strand cDNA Synthesis Kit (Takara, China). PCR was performed using 1 μl of the RT products as a template. For the amplification step, 40 cycles were conducted under the following conditions: denaturation at 94 °C for 2 min, annealing at 58 °C for 30 s, and extension at 72 °C for 45 s. The specific gene primers used for real-time PCR are presented in Table 1. Real-time PCR was used to measure the expression of the genes of Wnt (*MYC*, *CCND1*, *AXIN2*, *POU5F1*, *LRP5*) and β-catenin. All target gene expression results have been normalized to *ACTB*.

**Table 1:** Primers for quantitative real-time polymerase chain reaction analysis

Gene	Product size (base pairs)	Sequences
*MYC*	189	Forward: 5′-TCCTTTATCACACCCCCTACC-3′ Reverse: 5′-TTAGCCAAATTGTTCCCTGC-3′
*CCND1*	94	Forward: 5′-CCGAGAAGCTGTGCATCTACAC-3′ Reverse: 5′-AGGTTCCACTTGAGCTTGTTCAC-3′
*AXIN2*	112	Forward: 5′-GGCTCCAGAAGATCACAAAG-3′ Reverse: 5′-TATGGAATTTCTTCCCCACA-3′
*POU5F1*	115	Forward: 5′-AGCAAAACCCGGAGGAGT-3′ Reverse: 5′-CCACATCGGCCTGTGTATATC-3′
*LRP5*	138	Forward: 5′-GACCCAGCCCTTTGTTTTGAC-3′ Reverse: 5′-TGTGGACGTTGATGGTATTGGT-3′
β-catenin	77	Forward: 5′-AAGTGGGTGGTATAGAGGCTCTTG-3′ Reverse: 5′-GATGGCAGGCTCAGTGATGTC-3′
*ACTB*	108	Forward: 5′-TTGCTGACAGGATGCAGAAG-3′ Reverse: 5′-TAGAGCCACCAATCCACACA-3′

### Western blot analysis

Cells were washed with ice-cold PBS twice and lysed in high-salt lysis buffer (45 mM HEPES (pH 7.5), 400 mM NaCl, 1 mM EDTA, 10% Glycerol, 0.5% NP40, 6.25 mM NaF, 20 mM β-glycerophosphate, 1 mM DTT, 20 mM sodium butyrate, and 1× protease inhibitor cocktail; Roche). Equal amounts of protein extracts were resolved via 12% SDS-PAGE, and then transferred to a polyvinylidene difluoride membrane (BIO-RAD). The membranes were blocked with 5% (wt/vol) dry milk in PBS-Tween-20 (0.5% vol/vol) and probed with primary antibodies overnight. Anti-β-catenin (#9562) and anti-LRP5 (#5731) antibodies were purchased from Cell Signaling Technology (CST, USA). Secondary antibodies were conjugated to horseradish peroxidase (#7074; CST, USA) for 1 h at room temperature. The immunoreactivity was detected with an ECL kit (#6883; CST). Anti-ACTB antibodies (sc-47,778; Santa Cruz Biotechnology) were used as loading control. The quantification of western blot analysis was ushered by ImageJ software.

### Statistical analysis

Data were collected from three separate experiments and are shown as the mean ± standard deviation (SD). Statistical differences were analyzed with one-way analysis of variance (ANOVA) using SPSS 17.0. *p* < 0.05 was considered statistically significant.

## Results

### Characterization of 60 nm AuNPs

**Fig. 1:**
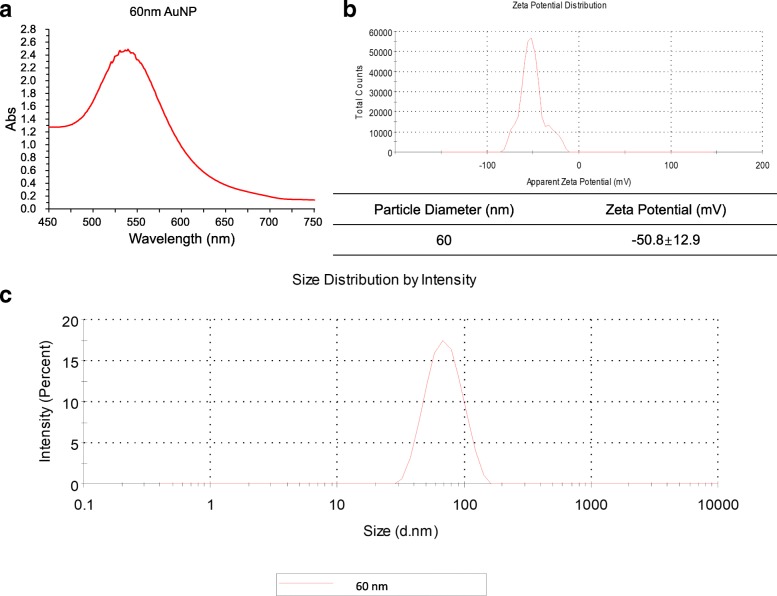
Size and zeta potential of 60 nm AuNPs. **a** UV–Vis absorption spectrum of AuNPs. **b** Zeta potential. **c** Size distribution, measured via DLS. Abs absorbance, AuNP gold nanoparticle

The 60 nm AuNPs peaked at 538 ± 14 nm (Fig. 1a). The Zetasizer showed that the size of AuNPs was 58.71 ± 22.33 nm (Fig. 1c) and the zeta potential was − 50.8 ± 12.9 mV (Fig. 1b).

#### Cell viability of hPDLSCs treated with β-catenin siRNA or LRP5 shRNA plasmid and cultured with AuNPs

**Fig. 2:**
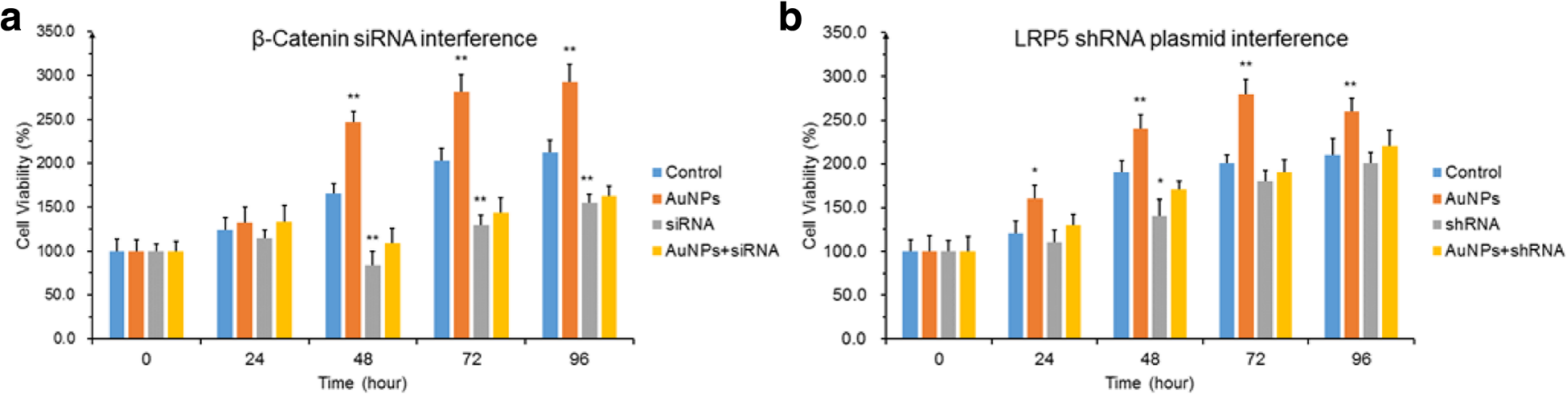
Cell viability of hPDLSCs. **a** hPDLSCs treated with AuNPs, β-catenin siRNA, or AuNPs in combination with siRNA. **b** LRP5 shRNA plasmid either in combination with AuNPs or without; hPDLSCs cultured only with AuNPs significantly higher in proliferation after 48 h compared to 96 h. hPDLSCs treated with serum-free medium as control in both assays. *p < 0.05, **p < 0.01. AuNP gold nanoparticle, shRNA short hairpin RNA, siRNA small interfering RNA

To evaluate the proliferation of hPDLSCs in the cell viability assay, hPDLSCs were cultured with AuNPs, β-catenin siRNA, or AuNPs and β-catenin siRNA (Fig. 2a), or with LRP5 shRNA plasmid or AuNPs and LRP5 shRNA plasmid (Fig. 2b). hPDLSCs treated by AuNPs profoundly increased in proliferation after 48 h of incubation and reached the plateau phase after 96 h of incubation. In addition, β-catenin siRNA significantly decreased the cell viability after 48 h of treatment regardless of whether AuNPs were added. The LRP5 shRNA plasmid did not profoundly impact hPDLSCs compared to the control, except in the 48-h treatment.

#### β-Catenin in the Wnt signaling pathway after blocking LRP5 by treating hPDLSCs with shRNA plasmid

**Fig. 3:**
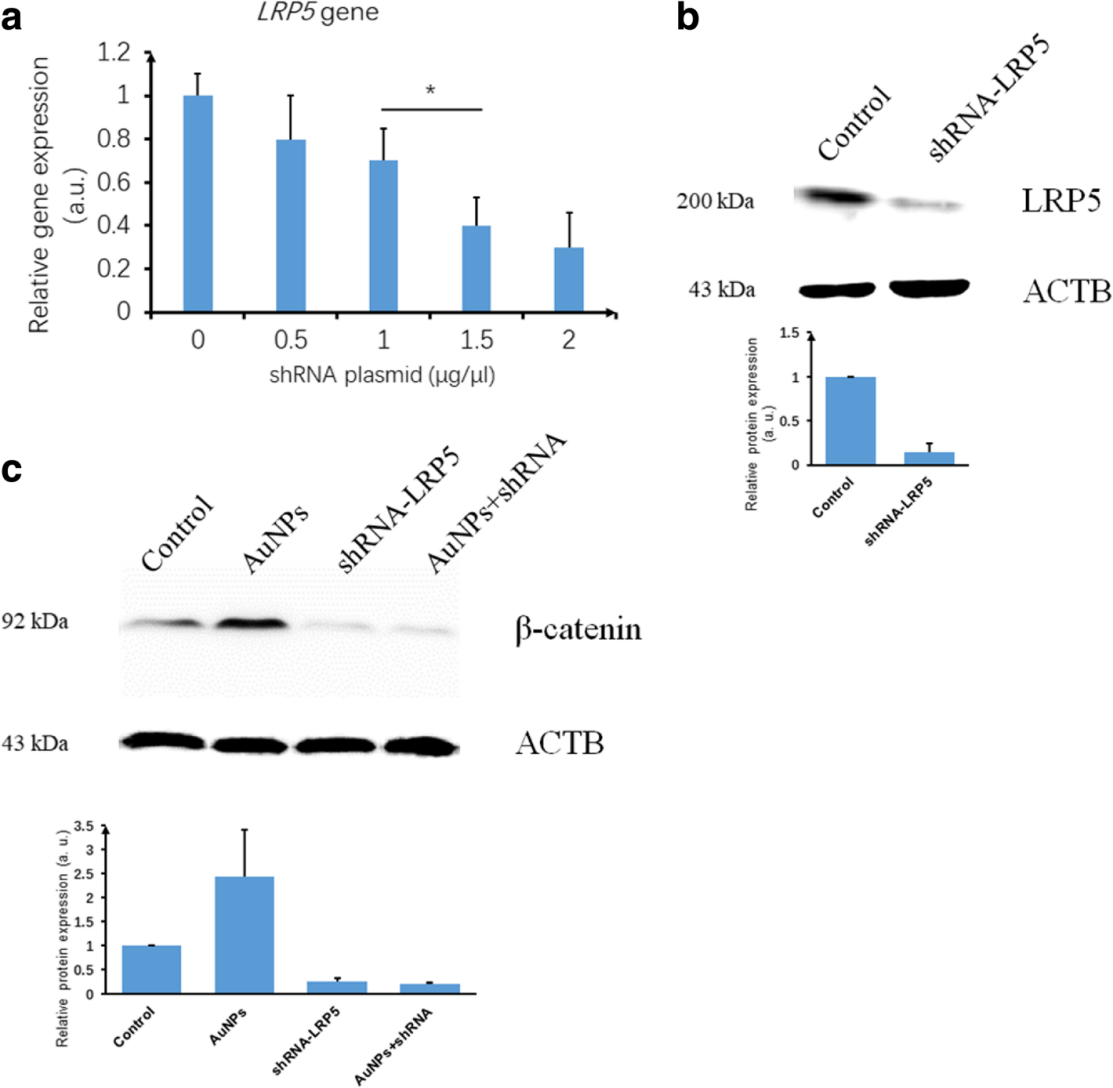
*LRP5* gene expression of hPDLSCs after interference with shRNA plasmid and β-catenin protein level after disturbance with shRNA plasmids. **a** *LRP5* gene expression after interference of different concentrations of shRNA plasmid. **b** LRP5 protein level after disturbance with 1.5 μg/μl shRNA plasmids. **c** β-catenin protein detection after shRNA plasmid and/or AuNP treatment. *p < 0.05. AuNP gold nanoparticle, a.u. arbitrary units, shRNA short hairpin RNA

The *LRP5* gene expression was detected via real-time PCR after interference with shRNA plasmids. Both 1.5 and 2.0 μg/μl concentration of the plasmid shRNA significantly downregulated the expression of the LRP5 gene (*p* < 0.05). No significant difference was found between 1.5 and 2.0 μg/μl (Fig. 3a). However, compared to 1.0 μg/μl shRNA, 1.5 μg/μl had a profound effect. Therefore, 1.5 μg/μl shRNA plasmid was used for further experiments. Western blot assay results (Fig. 3b) showed that, compared to the control group, 1.5 μg/μl shRNA plasmid significantly downregulated the expression of LRP5 protein.

Results showed that β-catenin significantly increased after incubation with AuNPs (60 nm at 56 μM) compared to the control group. Protein β-catenin of hPDLSCs did not increase after shRNA plasmid interference with LRP5 and even incubation with AuNPs, compared to the control group (Fig. 3c).

#### Blocking of β-catenin via treating hPDLSCs with siRNA

**Fig. 4:**
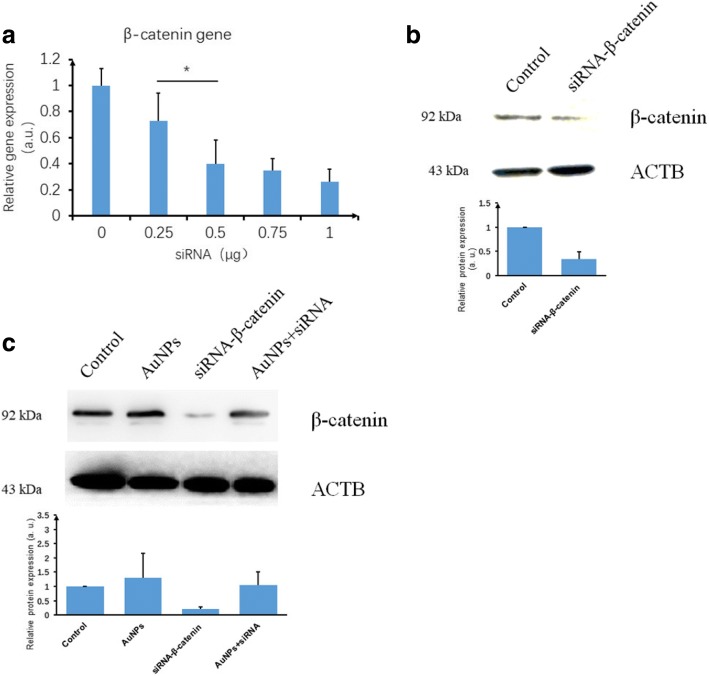
β-catenin gene expression after interference with siRNA. **a** Different concentrations of siRNA interfere with β-catenin gene expression in hPDLSCs. **b** β-catenin protein expression after disturbance with 0.5 μg/μl siRNA. **c** β-catenin protein detection after siRNA and/or AuNP treatment. *p < 0.05. AuNP gold nanoparticle, a.u. arbitrary units, siRNA small interfering RNA

siRNA 0, 0.25, 0.5, 0.75, or 1 μg was added to a 100 μl culture system. According to the results (Fig. 4a), 0.25, 0.5, 0.75, and 1 μg siRNA interfered with β-catenin expression, of which 0.5 μg siRNA significantly lowered β-catenin expression (*p* < 0.05) compared to 0.25 μg siRNA, while 0.5, 0.75, and 1 μg siRNA did not significantly downregulate β-catenin expression. Therefore, we chose 0.5 μg siRNA for further experiments. According to the results of western blot analysis (Fig. 4b) and compared to the control group, 0.5 μg siRNA significantly lowered β-catenin protein levels. Compared to the control, 0.5 μg siRNA profoundly decreased the β-catenin protein level, even under AuNP addition. The AuNP group had no significant impact on the β-catenin protein level (Fig. 4c).

#### Expression of *MYC*, *CCND1*, *AXIN2*, and *POU5F1*

**Fig. 5:**
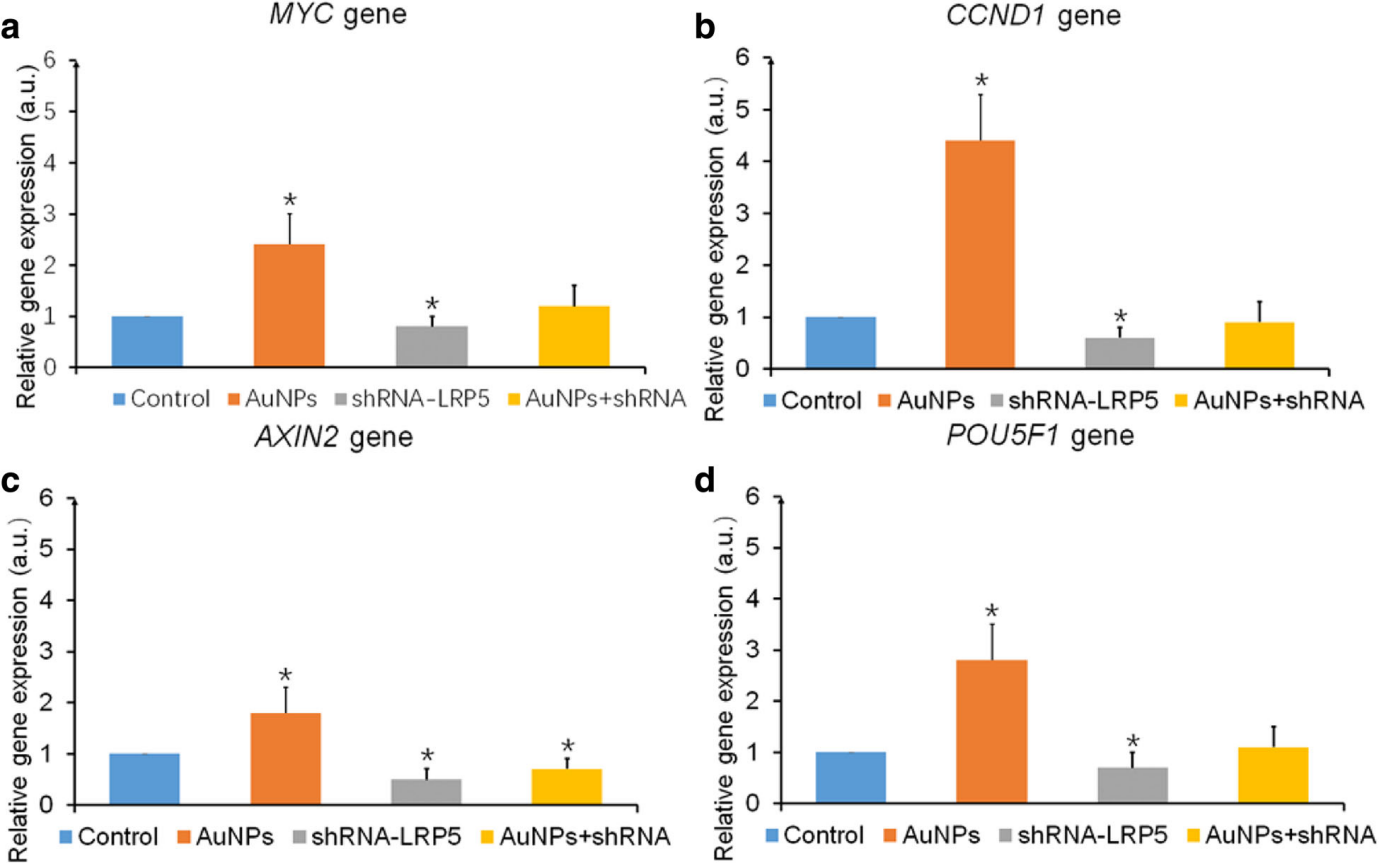
*MYC*, *CCND1*, *AXIN2*, and *POU5F1* gene expression after shRNA plasmids interfered with *LRP5* gene expression. *MYC* (**a**), *CCND1* (**b**), *AXIN2* (**c**), and *POU5F1* (**d**) gene expression after treatment with AuNPs (60 nm at 56 μM), shRNA-LRP5 plasmid, or both. *p < 0.05. AuNP gold nanoparticle, a.u. arbitrary units, shRNA short hairpin RNA

AuNPs (60 nm at 56 μM) and/or 1.5 μg shRNA plasmid were added to the hPDLSC culture system and the expressions of Wnt-related gens *MYC* (Fig. 5a), *CCND1* (Fig. 5b), *AXIN2* (Fig. 5c), and *POU5F1* (Fig. 5d) were detected via real-time PCR. AuNPs significantly increased the expression of *MYC*, *CCND1*, *AXIN2*, and *POU5F1* genes compared to control (*p* < 0.05). Interfering in the *LRP5* gene expression with shRNA plasmids downregulated expression of these four genes (*p* < 0.05). Under combined AuNP and shRNA plasmid treatment, only *AXIN2* gene expression was downregulated compared to control (*p* < 0.05).

**Fig. 6:**
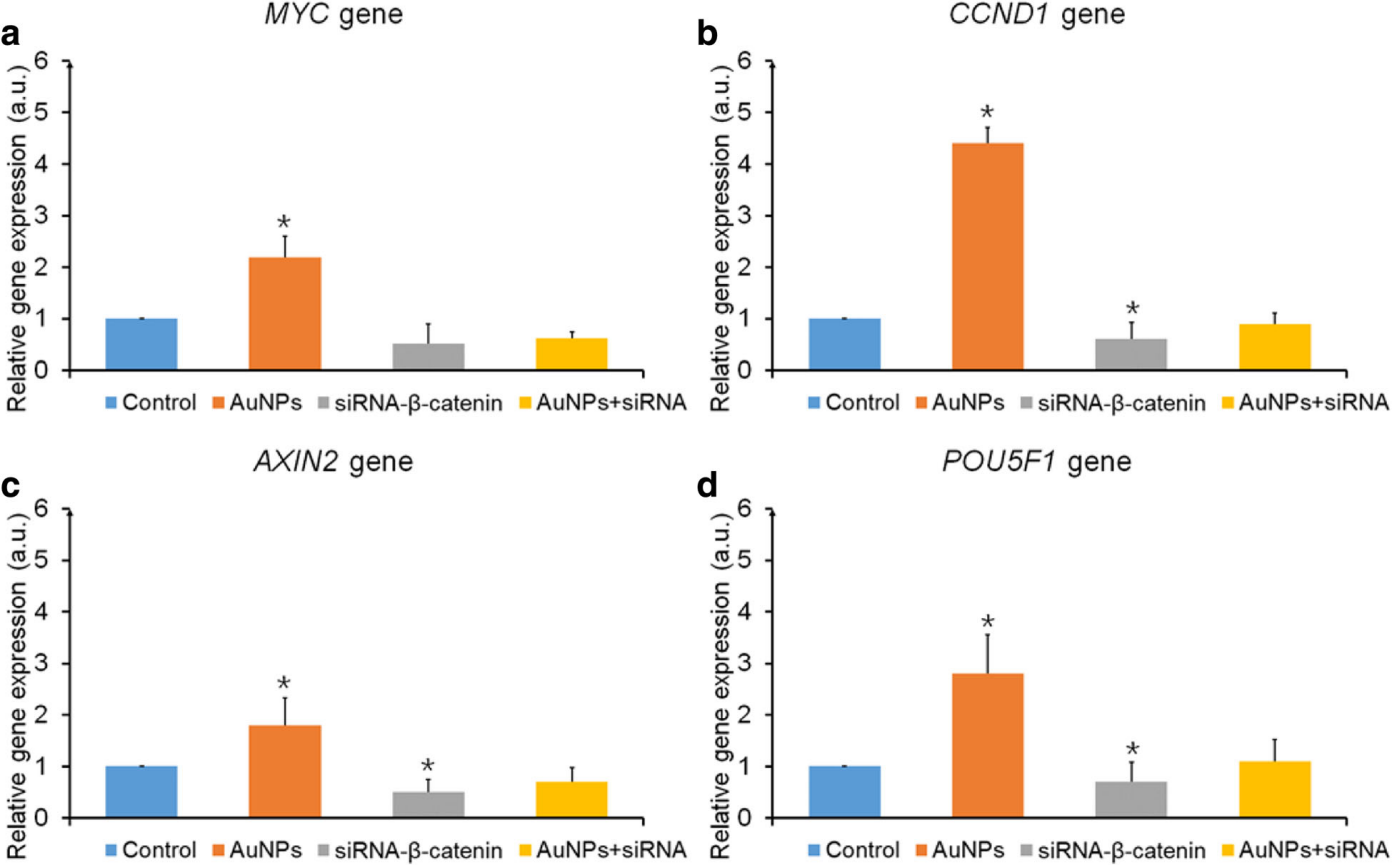
*MYC*, *CCND1*, *AXIN2*, and *POU5F1* gene expression after siRNA interference of β-catenin gene expression. *MYC* (**a**), *CCND1* (**b**), *AXIN2* (**c**), and *POU5F1* (**d**) gene expression after treatment with AuNPs (60 nm at 56 μM), siRNA-β-catenin, or both. *p < 0.05. AuNP gold nanoparticle, a.u. arbitrary units, siRNA small interfering RNA

AuNPs (60 nm at 56 μM) and/or 0.5 μg siRNA to β-catenin were employed to investigate the resulting effects on hPDLSCs in 100 μl medium per well and the Wnt/β-catenin signaling pathway-related genes *MYC* (Fig. 6a), *CCND1* (Fig. 6b), *AXIN2* (Fig. 6c), and *POU5F1* (Fig. 6d) were also detected via real-time PCR. AuNPs upregulated the expression of four genes significantly (*p* < 0.05). β-catenin was interfered with by siRNA and, except for *MYC*, the other three genes were downregulated (*p* < 0.05). Although β-catenin was part of the interference of siRNA, addition of AuNPs decreased the interference compared to the control.

## Discussion

Recently, many in-vitro and in-vivo studies have indicated that AuNPs are nontoxic; however, other publications contradict these results [7, 8, 15]. In spite of this, most studies focus on the pathophysiology and not on the physiology of AuNPs. Multiple in-vitro studies have shown that AuNPs at a certain particle size and concentration promote cell proliferation [4, 10]. Our previous experiments confirmed that 60 nm AuNPs at a concentration of 56 μM significantly promote the proliferation of human periodontal ligament cells and hPDLSCs [5]. However, the precise mechanism controlling the proliferation process still remains poorly understood.

AuNPs (60 nm, NIST standard reference material) in murine macrophages indicated no cytotoxicity under the exposure conditions tested, and neither do these elicit proinflammatory responses; however, the localization of AuNPs in intracellular vacuoles suggests endosomal containment and an uptake mechanism involving endocytosis [6]. The suggested size scale of AuNPs of 30–74 nm was presumed to facilitate internalization. AuNP sizes via DLS were 6.3 ± 2.5 nm in GNP-S, 18.1 ± 2.1 nm in GNP-M, and 23.0 ± 10.2 nm in GNP-L, respectively. The GNP-S showed significant cytotoxicity by ROS; however, the GNP-L exerted little toxicity. Therefore, AuNPs sizes exceeding 40 nm tend to show no cytotoxicity [16]. Considering the uptake efficacy was not increasing with AuNP size, AuNP diameters not exceeding 70 nm were chosen accordingly.

**Fig. 7:**
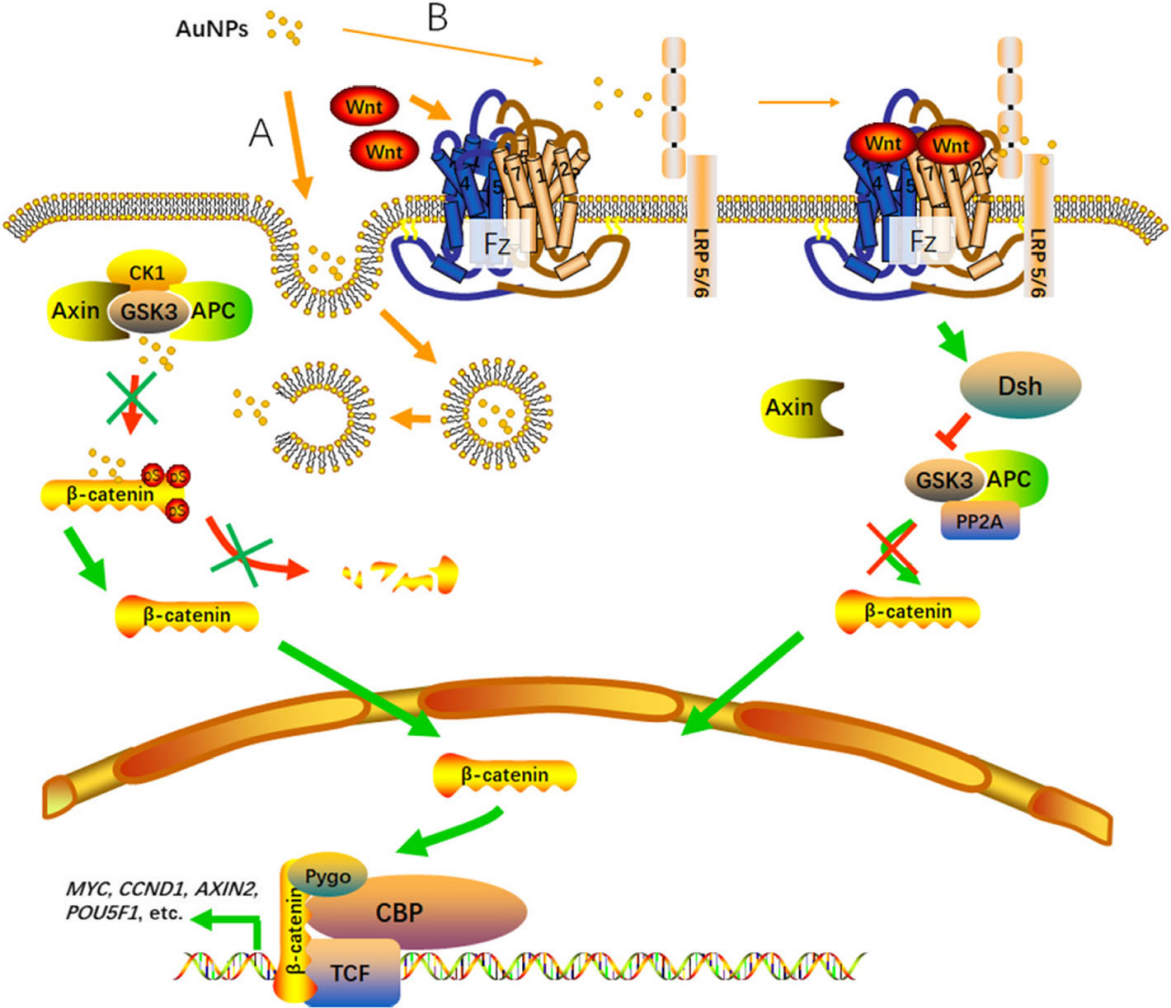
Scheme of AuNPs to Wnt/β-catenin signaling pathway in cell proliferation. **a** AuNPs enter cells via internalization to inhibit destruction of the complex (Axin, APC, PP2A, GSK3, and CK1α) to increase accumulation of β-catenin in both nucleus and cytoplasm. **b** AuNPs may help binding of Wnt, Fz, and LRP 5/6. Dsh is then activated via phosphorylation and inhibits GSK3 activity of the destruction complex. β-catenin released and localized in nucleus. Subsequently, β-catenin translocated into nucleus where it acts as a transcriptional coactivator of transcription factors of the TCF family and recruits histone modifiers such as CBP and Pygo. β-catenin influences target gene transcription. APC adenomatosis polyposis coli, AuNP gold nanoparticle, CK1 casein kinase 1, Dsh disheveled, Fz Frizzled, GSK3 glycogen synthase kinase 3, LRP lipoprotein receptor-related protein, PP2A protein phosphatase 2A, Pygo pygopus, TCF T-cell factor, CBP CREB (cAMP response element binding protein) binding protein

AuNPs with different surface charge were employed to treat human bone marrow-derived mesenchymal stem cells (hMSCs). Surface-functionalized AuNPs were without acute toxicity. Positively charged AuNPs showed higher cellular uptake. AuNPs did not inhibit osteogenesis but ALP activity and calcium deposition were remarkably reduced in response to negatively charged AuNP treatment. The proteins in the biological medium were likely coating AuNPs, leading to a change of the initial surface charge of nanoparticles [7]. Therefore, the initial surface charge was not sufficiently convincing as a simple predictor of nanoparticle uptake and cytotoxicity. Therefore, internalization was considered a reasonable route by which AuNPs enter cells [17] (Fig. 7).

Multiple signaling pathways, including SMAD, Notch, FGF, Hedgehog, and Wnt, have been closely associated with cell proliferation. Many related signaling pathways of stem cell proliferation have an intrusive cross-talk with Wnt signaling*.* Hence, the variation of the canonical Wnt signal pathway for the proliferation of hPDLSCs treated with AuNPs was investigated in this experimental study. Furthermore, AuNPs of size 30–74 nm had an uptake half-time of about 2 h, while the removal half-time was about 0.5 min, depending on cell types. Global gene expression analysis [18] and proteomic analysis [19] of AuNPs to human dermal fibroblasts indicated that the signaling pathways were inferred to respond during a short time. The Wnt signaling pathway was a robust candidate for the mechanism of AuNPs for promoting cell proliferation [11, 13]. This study showed that the proliferation of hPDLSCs boosted by AuNPs was significantly interfered with by β-catenin siRNA and slightly by LRP5 shRNA plasmid (Fig. 2). The Wnt signaling pathway was thus suggested as an important mechanism with which AuNPs promote the growth of hPDLSCs.

Our experimental results showed that the AuNPs could activate LRP5 and/or β-catenin, and then activate downstream stem cell proliferation-related genes such as *MYC*, *CCND1*, *AXIN2*, and *POU5F1*. Interfering with the gene expressions of *LRP5* and *β-catenin* with shRNA plasmid and siRNA respectively, the expressions of the aforestated genes were downregulated. After interfering with the gene expressions of *LRP5* and *β-catenin* with shRNA plasmid and siRNA, AuNPs were added and β-catenin protein did not increase. The expressions of the four tested genes were downregulated. The possible mechanisms with which AuNPs promote proliferation of human periodontal ligament stem cells were as follows: AuNPs outside the cells could promote LRP5, Wnt, and Frizzled protein aggregation and act on β-catenin in the cytoplasm. Phosphorylated β-catenin was dephosphorylated, transferred into the nuclei, and the expression levels of intracellular hPDLSC proliferation-related genes such as *MYC*, *CCND1*, *AXIN2*, and *POU5F1* increased, thus promoting hPDLSC proliferation.

When Wnt/β-catenin signaling pathways were activated, β-catenin accumulated in the cytoplasm, entered the nucleus, and activated the downstream gene *CCND1*. A protein of the cell cycle, *CCND1* regulates cells via G1 to S phase transformation, cell proliferation, and differentiation. Studies have shown that *CCND1* expression and its upregulation have a close relationship with activated Wnt signaling pathways and increase the β-catenin expression level [20]; however, this is still disputed [21]. β-catenin proteins and TCF-4 activate *MYC* after combination [22], thus regulating the proliferation and differentiation of stem cells.

*POU5F1* (also called *Oct4*) belongs to the family of POU and plays an important role in the maintenance of self-renewal of stem cells. In addition to the differentiation of mature stem cells, the expression of *POU5F1* gradually declines [23]. Therefore, its relative expression is very low in mature somatic cells in vivo and in vitro. The activation of Wnt signaling pathways promotes the expression of *POU5F1*. *AXIN2* participates in a β-catenin degradation compound. The *AXIN* family has two members: *AXIN1* and *AXIN2*. *AXIN2* has a very sensitive negative feedback effect on the Wnt signaling pathway. Whether *AXIN2* expression increases is an important indicator of whether the Wnt signaling pathway is involved in the regulation of cellular physiological and pathological physiology change. *AXIN2* as a Wnt signaling pathway inhibitory factor in the cell may regulate Wnt signal activation, where it leads to cell proliferation, prevents excessive cellular proliferation, and prevents malignant transformation [24, 25].

Cell proliferation is a complex process in which many factors interact. Our results suggest that AuNPs promote hPDLSC proliferation effectively, which is closely related to the Wnt/β-catenin signaling pathway; however, their relationship and the underlying mechanism have yet to be further revealed. Further studies of AuNPs on the hPDLSC proliferation effect and the underlying mechanism will not only allow us to better understand AuNPs and their interaction with hPDLSCs, but can also provide excellent advancements of periodontal tissue engineering and thus lead to good seed cells.

## Conclusion

In a previous experiment, we suggested that AuNPs (60 nm at 56 μM) efficiently promote the proliferation of hPDLCs/hPDLSCs in vitro. However, the AuNP-induced proliferation-boosting effect on hPDLSCs was diminished as β-catenin siRNA or LRP5 shRNA plasmid was added. The AuNP treatment equalized the accumulation of β-catenin in hPDLSCs. The proliferation of hPDLSCs was initiated by the Wnt/β-catenin signaling pathway. Nevertheless, more details of the interaction between AuNP proliferation and the Wnt/β-catenin signaling pathway are required to suggest more potential applications.

## Abbreviations

AuNP: Gold nanoparticle
BBI: British Biocell International
FCM: Flow cytometry
hMSC: Human bone marrow-derived mesenchymal stem cell
hPDLC: Human periodontal ligament cell
hPDLSC: Human periodontal ligament stem cell
